# Effect of Hemifacial Spasm on Intraocular Pressure Measurement

**DOI:** 10.1155/2018/3621215

**Published:** 2018-02-06

**Authors:** Erdogan Cicik, Rengin Yildirim, Ceyhun Arici, Funda Dikkaya, Osman Sevki Arslan

**Affiliations:** ^1^Department of Ophthalmology, Cerrahpasa School of Medicine, Istanbul University, Istanbul, Turkey; ^2^Department of Ophthalmology, Medipol University Medical Faculty, Istanbul, Turkey

## Abstract

**Purpose:**

To evaluate the effect of hemifacial spasm (HFS) on intraocular pressure (IOP) measurement.

**Methods:**

Twenty-four consecutive patients with HFS and 25 age- and gender-matched randomly selected eyes of healthy volunteers underwent corneal pachymetry and IOP measurements using Goldmann applanation tonometer (GAT) and noncontact tonometer (NCT). IOP measurements were performed before (during HFS) and 2 weeks after Botox injections in HFS patients and in healthy volunteers without Botox injections.

**Results:**

There was no statistical difference between involved eye side and uninvolved eye side of HFS patients in measured central corneal thickness. Similarly, no difference was found between involved eye side of HFS patients and controls. There were no statistically significant differences comparing IOP values before treatment and levels measured at 2 weeks of Botox injections, either with GAT (*p* = 0.33, 0.11) or NCT (*p* = 0.80, 0.43) devices in the involved eyes and uninvolved eyes of patients with HFS, respectively. There were also no significant differences in these parameters (GAT (*p* = 0.63) and NCT (*p* = 0.54)) in controls.

**Conclusions:**

Contractions in facial muscles may not lead to significant increase in IOP in HFS patients. This result may help clinical decision making in the treatment of glaucoma patients with HFS. This trial is registered with NCT03390803.

## 1. Introduction

Hemifacial spasm (HFS) is characterized by unilateral intermittent tonic or clonic contractions in the facial musculature innervated by the facial nerve. Usually, its onset occurs in the 5th and 6th decades, is unilateral, and is caused by nerve compression at its exit root by an aberrant or deviated arterial vascular branch [1]. HFS is a chronic disorder and may have a severe impact on the patient's appearance. Unlike blepharospasm, hemifacial spasm persists during sleep and is unrelated to hypersensory input. It may lead to insomnia [2, 3]. Spontaneous remission is extremely rare [4]. There is no known cure for HFS. The best available treatment is repeated botulinum neurotoxin (BoNT) injections [4]. BoNT is an exotoxin produced by the bacterium *Clostridium botulinum*, an anaerobic Gram-positive sporulating organism [5].

Intraocular pressure (IOP) is the most important and only modifiable risk factor in patients with glaucoma. Accurate IOP measurement plays a crucial role in diagnosis as well as management of glaucoma [6]. Numerous factors influence the IOP measurement, especially central corneal thickness (CCT), corneal curvature, scleral rigidity, patient positioning at the slit lamp, direction of gaze [7], and the technique used for the measurement. The most reliable method for the IOP measurement is based on the application principle. The Goldmann applanation tonometer (GAT) and noncontact tonometer (NCT) both use an applanation principle.

Physiologic conditions (eye movement, blinking, or eyelid squeezing) are declared to cause IOP variations. Coleman and Trokel [8] reported that efforts at accommodation resulted in an IOP increase of 2 to 4 mmHg despite cycloplegia. In the same study, eyelid closure produced an IOP increase of 5 mmHg, eyelid blinking an increase of 5 to 10 mmHg, and forced eyelid squeezing an increase of 90 mmHg. Extreme upgaze produces the greatest IOP elevation of all eccentric eye positions (mean: 6.8 mmHg) [9].

Since there is increased frequency of repeated eyelid blinking and forceful eyelid closure in HFS patients, we suggested that IOP measurements could be affected by these characteristics in this group of patients. To our knowledge, the relationship between HFS and IOP measurement has not been investigated. The current study was conducted to evaluate the effect of HFS on IOP measurement using GAT and NCT.

## 2. Material and Methods

The study protocol was approved by the Ethics Committee of Istanbul University Cerrahpasa Faculty of Medicine and conducted in accordance with the tenets of the Helsinki Declaration. Informed consent was obtained from all patients and healthy volunteers participating in the study. Twenty-four consecutive patients with HFS (study group) and twenty-five age-matched and gender-matched healthy volunteers (control group) were prospectively included in the study. In the control group, selection of side to include in the study was based on random selection.

Each patient underwent a full ophthalmological examination, including slit lamp evaluation, stereoscopic optic disk examination, and normal achromatic automated perimetry. We excluded any cases with a previous glaucoma diagnosis (i.e., vertical cup-to-disc ratio of >0.6, cup-to-disc asymmetry of >0.2, the presence of neuroretinal rim thinning or notching, peripapillary hemorrhages, or nerve fiber layer defect). All injections were performed subcutaneously by the same physician (R. Y.). Botox (Allergan Inc., Irvine, CA, USA) was used in all cases. The manufacturer's instructions were followed. BoNT was diluted with 2 ml of sterile, preservative-free saline solution to obtain 5 units in 0.1 ml and injected within 10 minutes of reconstitution. Inclusion criteria included patients who were 20 years of age or older with the diagnosis of HFS, who were not treated with BoNT injections before. Subjects were excluded if they had allergies to botulinum toxin or any component of the drug; previous eyelid, refractive, or intraocular surgery; any abnormality preventing reliable tonometry in either eye; strabismus; contact lens wear; pregnancy; glaucoma; and ocular hypertension and are using agents that could interfere with neuromuscular transmission.

IOP measurements were performed before and 2 weeks after Botox injections in patients with HFS (both involved and uninvolved eyes with HFS) and in healthy volunteers without Botox injections. Patients were seated comfortably in an examination chair during all measurements. An interval of 15 minutes was maintained between GAT and NCT measurements.

All the measurements with GAT were done by a single physician (E. C.) while those with the NCT were performed by another physician (C.A.) who was masked to the results of the GAT. All measurements were taken between 10:00 and 11:00 a.m. The IOP was measured in primary gaze holding the eyelids open against the orbital rim, in both patient and control groups. The mean of three NCT measurements was recorded in each eye. The IOP was measured using GAT by the following protocol [10, 11]: patients received a drop of 0.25% fluorescein with 0.5% proparacaine in each eye; 2 consecutive GAT measurements were made; if the difference between the two measurements was ≤2 mmHg, then the average of these measurements was recorded for the correspondent eye. If the difference between two measurements was >2 mmHg, then another measurement was made and the median of the three measurements was recorded. The central corneal thickness (CCT) was measured with an ultrasound pachymeter (US 4000, Nidek, Japan).

### 2.1. Statistical Analysis

The data were statistically analyzed with IBM SPSS Statistics Standard Pack 21, Licensing Type: Network, Istanbul University Licensed Software. Data normality was assessed using the Kolmogorov-Smirnov test. Continuous variables with normal distribution were compared by using Student's *t*-test, presenting the results as mean and standard deviation. *p* < 0.05 was considered statistically significant.

## 3. Results

The mean age was 45.3 ± 9.6 years (16 female, 8 male) for the cases with HFS and 44.4 ± 9.7 years (16 female, 9 male) for the control group (*p* = 0.77). 12 patients with HFS had right hemiface involvement and 12 patients had left hemiface involvement.

There was no statistical difference between involved eye side and uninvolved eye side of HFS patients in terms of CCT measurements (mean CCT: 534.8 ± 27.7 and 535.6 ± 28.2, respectively, *p* = 0.92). The mean CCT thickness was 543.8 ± 24.8 in control groups. Similarly, no difference was found between involved eye side of HFS patients and controls (*p* = 0.62).

In the study group, the average pretreatment IOP levels were 14.5 ± 1.8 with GAT and were 16.2 ± 1.5 with NCT device. These levels were measured as 14.3 ± 1.9 with GAT and 16.2 ± 1.9 with NCT at second week visit after injections. The IOP measurements taken before Botox injections and at 2 weeks of Botox were not different for GAT (*p* = 0.33) and NCT (*p* = 0.80) in patients with HFS (Table 1). There were also no significant differences in these parameters in the uninvolved eyes of patients with HFS and healthy volunteers (Tables 2 and 3).

## 4. Discussion

Reliable measurement of IOP is important for the management and follow-up of patients with glaucoma. IOP is the only measurable risk factor. There are numerous sources of error that can lead to inaccurate IOP readings; they include measuring procedure, anatomical changes of the eye (corneal thickness, corneal diseases, and scleral rigidity), or extraocular influences (direction of gaze, reflex tearing, examiner's pressure to hold the eyelids open, and Valsalva maneuver) [12–18]. Due to the fact that IOP measurements are affected by CCT values, we aimed to measure CCT in the study and control groups to comment IOP measurement results according to CCT results of groups. No difference was found between involved eye side and uninvolved eye side of HFS patients and involved eye side of HFS patients and healthy volunteers in terms of CCT measurements.

In a study evaluating the effect of attempted eyelid closure on IOP measurement in normal subjects, the mean increase in IOP associated with forcible attempted eyelid closure by the healthy subjects was found to be statistically significant using both GAT and the electronic Tono-Pen XL. Increases in IOP up to 8 to 9 mmHg occurred in some subjects [12]. Jamal et al. [13] showed that the mean increase in IOP associated with forced attempted eyelid closure is even greater in glaucoma patients, with increases up to 11 to 14 mmHg.

In a different research, Killer et al. [19] reported on a case with HFS and glaucomatous optic atrophy. Miller performed readings of the IOP during blinks and found an average pressure change of 10.3 mmHg. During a hard lid squeeze, the pressure can rise to 51 mmHg [20]. Coleman and Trokel [8] used an invasive method by placing a 23-gauge needle connected to a pressure monitor into the anterior chamber of a volunteer's eye with choroidal melanoma that was going to be enucleated for pressure readings and found IOP spikes of up to 90 mmHg during forced lid squeeze. Considering the high frequency of clonic and tonic episodes of lid squeezes in patients with HFS, it seems at least probable that glaucomatous damage might occur over time due to the pressure that is generated during periods of orbicularis contractions.

Green and Luxenberg [21] studied normal subjects and 19 patients with either ocular hypertension or glaucoma to determine the effect of forcible eyelid squeezing on IOP. They used an applanation tonometer to measure the pressures while the subjects were in the supine position. The procedure entailed having the subjects forcibly squeeze their eyelids for a period of 2 seconds and then rest for two seconds over a one-minute period. Results showed that there were two types of responders in the normal group. After 60 seconds of forcible eyelid squeezing, done for 2 seconds every 4 seconds, IOP decreased once the eyelid relaxed. In “responder” normal subjects, the decreased relaxation level remained from 5 to 10 minutes. In “nonresponder” normals, change in IOP was minimal. The study found that the decline in IOP following eyelid squeezing was significantly less in the glaucoma group than that in the normal responders. In contrast, the recovery of the presqueeze IOP was faster in the normal group than that in the glaucoma group. Involuntary eyelid squeeze may result in increased IOP in HFS patients with glaucoma and recovery of IOP following Botox injections may be affected, although no IOP difference was found in HFS patients without glaucoma before and after Botox injections.

The mean differences in IOP measurements taken before and after Botox injections in our study were not statistically significant in HFS patients with no glaucoma. This result may be valuable in the follow-up of glaucoma patients who also have HFS. Physicians might consider increased levels of IOP during the spasmodic HFS episodes as related to forcible blinks or eyelid spasms; however, according to the results of our study, HFS spasms do not lead to increased levels of IOP. However, even a small increase in IOP may have clinical significance in glaucoma. Results from the Early Manifest Glaucoma Trial suggest that even a 1 mmHg increase in IOP was associated with an 11% increase in the hazard ratio for the progression of glaucoma [22]. Thus, measuring IOP accurately is critical in the management of glaucoma, which is no different in patients with HFS. However, our study may not directly comment on the relation of IOP changes in HFS patients with glaucoma. Although our results indicate that the IOP is not affected in HFS patients before and after Botox injections, we may only suggest that this relation may be also true for the patients with actual glaucoma. Further studies in a study group including patients with HFS and glaucoma compared to control group consisting of patients with HFS and no glaucoma may give more insight about this hypothesis.

HFS is characterized by the spontaneous onset of unilateral intermittent spasms of the orbicularis oculi muscle. These spasms gradually increase in severity and frequency and spread downward to involve the muscles of facial expression, including the platysma. We did not find an increase in IOP in patients with HFS, although different studies showed increased IOP after forceful closure of eyelids [8, 12, 13, 20, 21]. Perhaps intermittent spasms of the orbicularis oculi muscle do not result in increase in IOP during measurement. Another thought is that the severity and frequency of the spasms are not enough to result in increase in IOP.

This study has some limitations. It included a relatively small study group population; therefore, statistical analysis may not be accurately interpreted. It was a cross-sectional study and did not include HFS patients with glaucoma.

In conclusion, although it has been reported that attempted forced eyelid closure by the normal subjects during tonometry increased IOP, in our study, involuntary facial muscle spasm in HFS did not cause increase in IOP. IOP measurement is important in the diagnosis and follow-up of glaucoma patients with HFS. We believe that the results of this study could help in the diagnosis, follow-up, and treatment of patients with ocular hypertension and glaucoma in patients with HFS when the IOP measurements are in the grey area.

## Figures and Tables

**Table 1:** Average intraocular pressure^a^ in the involved eyes of patients with hemifacial spasm before and after Botox injections using Goldmann applanation tonometry and noncontact air puff tonometry.

IOP	Before Botox injection	After Botox injection (at second week)	*p* value^b^
Goldmann	14.5 ± 1.8	14.3 ± 1.9	0.33
NCT	16.2 ± 1.5	16.2 ± 1.9	0.80

^a^Mean ± SD (mmHg). ^b^Paired samples *t*-test. NCT: noncontact tonometry; IOP: intraocular pressure.

**Table 2:** Average intraocular pressure^a^ in the uninvolved eyes of patients with hemifacial spasm before and after Botox injections to the involved side of the hemiface using Goldmann applanation tonometry and noncontact air puff tonometry.

IOP	Before Botox injection	After Botox injection (at second week)	*p* value^b^
Goldmann	14.7 ± 2.1	14.4 ± 1.8	0.11
NCT	16.3 ± 1.7	16.1 ± 1.8	0.43

^a^Mean ± SD (mmHg). ^b^Paired samples *t*-test. NCT: noncontact tonometry; IOP: intraocular pressure.

**Table 3:** Average intraocular pressure^a^ in the eyes of healthy volunteers with two weeks interval using Goldmann applanation tonometry and noncontact air puff tonometry.

IOP	First measurement	Second measurement (at second week)	*p* value^b^
Goldmann	15.2 ± 2.1	15.0 ± 1.5	0.63
NCT	16.0 ± 1.7	16.2 ± 1.5	0.54

^a^Mean ± SD (mmHg). ^b^Paired samples *t*-test. NCT: noncontact tonometry; IOP: intraocular pressure.
